# Can neonatal lung ultrasound monitor fluid clearance and predict the need of respiratory support?

**DOI:** 10.1186/cc11865

**Published:** 2012-11-14

**Authors:** Francesco Raimondi, Fiorella Migliaro, Angela Sodano, Angela Umbaldo, Antonia Romano, Gianfranco Vallone, Letizia Capasso

**Affiliations:** 1 Division of Neonatology, Department of Pediatrics, Università "Federico II" di Napoli, via Pansini 5, Naples, 80131, Italy; 2 Department of Radiology, Università "Federico II" di Napoli, via Pansini 5, Naples, 80131, Italy

## Abstract

**Introduction:**

At birth, lung fluid is rapidly cleared to allow gas exchange. As pulmonary sonography discriminates between liquid and air content, we have used it to monitor extrauterine fluid clearance and respiratory adaptation in term and late preterm neonates. Ultrasound data were also related to the need for respiratory support.

**Methods:**

Consecutive infants at 60 to 120 minutes after birth underwent lung echography. Images were classified using a standardized protocol of adult emergency medicine with minor modifications. Neonates were assigned to type 1 (white lung image), type 2 (prevalence of comet-tail artifacts or B-lines) or type 3 profiles (prevalence of horizontal or A lines). Scans were repeated at 12, 24 and 36 hours. The primary endpoint was the number of infants admitted to the neonatal ICU (NICU) by attending staff who were unaware of the ultrasound. Mode of respiratory support was also recorded.

**Results:**

A total of 154 infants were enrolled in the study. Fourteen neonates were assigned to the type 1, 46 to the type 2 and 94 to the type 3 profile. Within 36 hours there was a gradual shift from types 1 and 2 to type 3. All 14 type 1 and 4 type 2 neonates were admitted to the NICU. Sensitivity was 77.7%, specificity was 100%, positive predictive value was 100%, negative predictive value was 97%. Four type 1 infants were mechanically ventilated.

**Conclusions:**

In the late preterm and term neonate, the lung ultrasound scan follows a reproducible pattern that parallels the respiratory status and can be used as a predictor of respiratory support.

## Introduction

The fetal lung is filled with fluid actively secreted by the pulmonary epithelium on a chloride ion gradient [1]. At birth, the fluid is rapidly cleared to allow post-natal gas exchange through epithelial sodium channels [2]. Impairment of this transition has been linked to neonatal respiratory distress (RD), particularly after cesarean section [3]. A similar mechanism has also been postulated for the increased rate of RD in late preterm newborns [4].

At present, there are no good techniques to follow the gradual passage to an air filled lung of a newly born infant and much relies on clinical and radiological signs.

In an experimental animal model, Jambrik *et al. *found a close correlation between the dry/wet ratio in the minipig lung and the number of ultrasound lung comets also known as B-lines [5].

In adult medicine, lung ultrasound scan has been successfully used to monitor the reverse change, that is, from a dry to a wet lung [6]. This standardized, non-invasive technique can diagnose pulmonary edema with high sensitivity and specificity and no radiation exposure [7].

The present study explores the potential of ultrasound as a monitoring tool of fluid to air passage in the neonatal lung. Ultrasound data suggesting a deranged changeover are also correlated with the need for respiratory support. Our approach, then, differs from previous studies performed mostly on preterm babies looking at the sonographic diagnosis of specific causes of respiratory distress [8-10].

## Materials and methods

Normal lung tissue scanned with ultrasound yields the superficial image of the pleural line rhythmically moving (the lung sliding sign) and horizontal repetition artifacts known as 'A lines'. Fluid accumulation in the alveolar interstitial space generates 'B lines', that is, comet-like, vertical artifacts [11]. Lichtenstein and Meziere have standardized the study of the adult lung interstitial syndrome describing the prevalence of B lines when scanning the anterior and lateral chest wall. In their series, this 'B profile' with present lung sliding diagnosed pulmonary edema with 97% sensitivity and 95% specificity [7].

**Figure 1:**
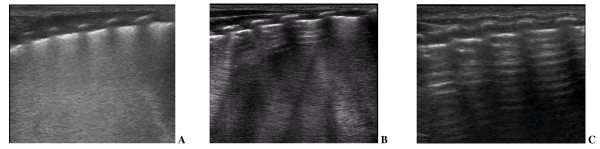
**Neonatal lung ultrasound profiles**. **A**) Type 1- full hyperechoic image of the lung fields or 'white lung'; **B**) Type 2- prevalence of B lines, that is, vertical, comet-tail artifacts; **C**) Type 3- predominance of A lines, that is, horizontal repetitions of the pleural line.

In the present study, we have modified the previous classification into three profiles:

Type 1 - full hyperechoic image of the lung fields or 'white lung' (Figure 1A); Type 2 - prevalence of B lines, lung sliding sign present (Figure 1B); Type 3 - A lines predominance, lung sliding sign present (Figure 1C).

The investigation was carried out at the well baby nursery and neonatal ICU (NICU) of the University "Federico II" of Naples, the largest regional delivery center with 2,400 live births/year. The study was approved by the local ethics committee (AOU "Federico II") and parental consent was obtained. As a general policy, newly born babies were brought to the Nursery. Infants were admitted to the NICU if signs and symptoms of RD (nasal flaring, expiratory grunting, tachypnea, shallow breathing, and so on) were present beyond the normal observation time of four hours; it was the responsibility of the attending physician, who was unaware of infants being scanned, to set the indication for respiratory support.

The purpose of the study was to evaluate the relative distribution of the three profiles and document the eventual transition from types 1 and 2 to type 3 with serial scans; to correlate the persistence of significant fluid as assessed by ultrasound with clinical outcome expressed as NICU admission rate, use of supplemental oxygen, nasal continuous positive airways pressure (N-CPAP) and mechanical ventilation. Clinical decisions were taken by a separate team of attending physicians, blinded to the study, operating according to the local NICU protocols. All live neonates with gestational age ≥34 weeks were included in the study: 112 (72.8%) full-term neonates (range 37 to 42 weeks) and 42 (27.2%) pre-term neonates (range 34 + 1 to 36 + 6 weeks); 120 (55%) had a birth weight greater than 2,500 grams, while 34 (22%) weighed less; 106 (68.8%) infants were delivered by cesarean-section while 48 (31.1%) were born vaginally; 107 (69.5%) babies were not given antenatal steroids whereas 40 (26%) received the treatment. Newborns with major congenital malformations and/or intrauterine growth retardation (IUGR) were excluded. Scans on the anterior and lateral chest walls of both lungs in supine infants were acquired in the Nursery by a single neonatologist (AS or FM) who then sent the acquired image to the Radiology Department in a separate building. The pediatric radiologist (GV) reviewed and classified the scans according to the study protocol. After completion of the study, concordance was assessed between the evaluation of the initial operator, who could not avoid observing the infant, and the pediatric radiologist who was fully masked to the infants' clinical conditions. Any clinical decision (NICU admission and type of treatment for respiratory distress) was taken by a third party physician, unaware of the present study. In our hospital, the Nursery and the NICU are separate environments attended by different medical staff. The infants were scanned in the absence of the physician in charge, taking extra care not to report the results of our investigation.

A broadband linear transducer (mod L12-5, Philips, Eindhoven, the Netherlands) which encompasses the superior and inferior lung fields in the same image was used. All infants had the first scan performed no sooner than one hour and no later than two hours after birth. Scans were then repeated at 12, 24 and 36 hours.

### Statistics

The study was conducted in a level III hospital with 2,400 total births per year. The population of term and late preterm infants was 2,200 with 11% admitted to the NICU for RD in 2010. We calculated that a cohort of 150 neonates would be representative of the population given a sampling error <5% with a confidence interval of 95%. When lung ultrasound images had been classified by the neonatologists and by the pediatric radiologist who was blind to the infants' clinical conditions, we investigated the accuracy of the sonographic profile with maximal echodensity (type 1 or white lung) to predict admission to the NICU for respiratory support.

We defined true positive (TP) as type 1 and admitted to the NICU; true negative (TN) as type 2 or 3 and not admitted; false positive (FP) as type 1 and not admitted; false negative (FN) as type 2 or 3 and admitted. The specificity of the test was defined TN/(TN + FP); sensitivity was TP/(TP + FN); positive predictive value (PPV) was TP/(TP + FP); negative predictive value (NPV) was TN/(TN + FN). Kappa coefficient was calculated to assess the interobserver variability.

## Results

One hundred fifty nine neonates were enrolled in the study. Two were excluded for congenital malformations (cystic adenomatous malformation and Tetralogy of Fallot) and three for IUGR. Population characteristics are described in Table 1. Based on the initial ultrasound scan, 14 neonates were assigned to type 1, 46 to type 2 and 94 to type 3 profiles. Review by the pediatric radiologist gave a full interobserver concordance (kappa = 1).

**Table 1:** Characteristics of the 154 subjects at the time of enrollment.

	Number (%)
Mode of delivery	
Vaginal delivery	48 (31.1)
C-Section	106 (68.8)
Gender	
Male	72 (46.8)
Female	82 (53.2)
Birth weight	
<2500 gr	34 (22)
≥2500 gr	120 (55)
Gestational age	
34 + 1/7 to 36 + 6/7 weeks	42 (27.2)
37 to 42 weeks	112 (72.8)
Use of antenatal steroids	
Yes	40 (26)
No	107 (69.5)
Unknown	7 (4.5)

Sequential scans in Table 2 show a gradual shift from type 1 and 2 to type 3 that was almost complete at 36 hours. As reported in Table 3, all 14 neonates initially classified as type 1 were admitted to the NICU (average age at admission: 5 hours) with a clinical diagnosis of RD (tachypnea, shallow breathing, grunting, nasal flaring). Among these, four infants with a 'ground glass' chest X-ray picture suggestive of hyaline membrane disease (HMD) received surfactant and were mechanically ventilated. The remaining ten neonates had an unremarkable chest X-ray and were supported with N-CPAP and supplemental oxygen.

**Table 2:** Lung ultrasound findings at sequential scans.

	Type 1	Type 2	Type 3
Initial scan	14/154	46/154	94/154
Within 12 hours	6/154	12/154	136/154
Within 24 hours	2/154	4/154	148/154
Within 36 hours	0/154	2/154	152/154

**Table 3:** Lung ultrasound findings and clinical treatment.

	Type 1	Type 2	Type 3
NICU admissions	14/14	4/46	0/94
Oxygen therapy	14/14	4/46	0/94
nCPAP	10/14	4/46	0/94
SIMV	4/14	0/46	0/94

nCPAP, nasal continuous positive airways pressure; NICU, neonatal ICU; SIMV: synchronized intermittent mandatory ventilation.

The four infants in the type 2 group (8.7%) who were admitted to the NICU with clinical RD and unremarkable chest X-ray received support with N-CPAP and supplemental oxygen. No baby in the type 3 group needed NICU admission or additional respiratory support. Given the above pattern distribution, we calculated the performance of the Type 1 profile in predicting NICU admission: sensitivity was 77.7%, specificity was 100%, PPV was 100% and NPV was 97%. Likewise, we calculated the accuracy of the Type 1 profile in identifying the four HMD/RDS cases (sensitivity = 100%; specificity = 93%; PPV = 28%; NPV = 100%). The remaining 14 infants admitted to the NICU were affected by transient tachypnea of the neonate (TTN) and the accuracy of the Type 1 profile was as follows: sensitivity = 71.4%; specificity = 97.1%; PPV = 71.4%; NPV = 97.1%.

## Discussion

This study shows that in the term and late preterm infant the early lung ultrasound profile can be classified in distinct patterns correlated to the lung fluid content. We document an evolution to the least hyperechogenic profile that is consistent with a clinically stable baby.

We found no previous approach using serial ultrasound scans to examine the extra-uterine respiratory adaptation in this neonatal population. Most other studies were focused on the preterm baby lung. In an unmasked investigation of 32 preterm infants, Copetti *et al. *described a specific sign of TTN comparing a single sonographic scan with the corresponding chest X-ray [8]. Using the same approach, they also studied the ultrasound appearance of surfactant deficiency in a group of 40 infants with a mean gestational age of 27 weeks compared to 15 significantly larger and more mature controls (mean gestational age: 30 weeks) [9]. In the very preterm population, respiratory distress syndrome is associated with a sonographic white lung image that remains unmodified after surfactant replacement [12].

Studying less immature neonates, we focused our attention on the clinical outcomes in our series and the practical implications for the clinical neonatologist regardless of nosologic classifications. When compared with neonates at 39 to 40 weeks of gestation, the risk of respiratory failure almost triples at 37 weeks and increases more than tenfold at 34 weeks. Similarly, there is a significant increase in the risk of developing symptomatic TTN (adjusted odds ratio 6.1 at 36 and 14.7 at 34 weeks, respectively) or hyaline membrane disease (adjusted odds ratio 9.1 at 36 and 40.1 at 34 weeks, respectively) [13].

As the regional organization of deliveries is often based on a 'hub and spoke' model, the timely identification of those infants in need of moderate to advanced respiratory support is crucial. Our results can help in this task. Likewise, an early type 3 profile (identical to the previously described normal lung image) was always confirmed at 12 hours and never associated with RD; this is also of clinical significance to those clinicians operating in birth centers where additional support equipment is not readily available.

We acknowledge some limitations of the present study. First, although an early allocation in the Type 1 group was effective in predicting the need for respiratory support, it did not discriminate between a milder and a more severe condition requiring mechanical ventilation. Second, the neonatologists acquiring the scans could not avoid observing the infant. This, however, was not critical in classifying the images, as assessed by the full agreement with the pediatric radiologist who was masked to the clinical conditions of the neonates.

## Conclusions

In the late preterm and term neonate, lung ultrasound scan follows a reproducible pattern that parallels the respiratory status and can be used as a predictor of the need for respiratory support. Health care givers working in a low technology setting can use lung ultrasound for early screening of neonates in need of respiratory support.

## Key messages

• At birth, lung fluid content can be assessed non-invasively by ultrasound.

• Over the first 36 hours, pulmonary adaptation can be monitored with serial ultrasound scans.

• Lung ultrasound permits the early and reliable identification of those newborns who fail to adapt and show signs and symptoms of respiratory distress.

• Health care givers working in a low technology setting can use lung ultrasound for an early screening of neonates in need of respiratory support.

## Abbreviations

HMD: hyaline membrane disease; IUGR: intrauterine growth retardation; NICU: neonatal intensive care unit; N-CPAP: nasal continuous positive airways pressure; NPV: negative predictive value; PPV: positive predictive value; RD: respiratory distress; TTN: transient tachypnea of the neonate.

## Competing interests

The authors declare that they have no competing interests.

## Authors' contributions

FR conceived the study design and wrote the manuscript. FM and AS performed the ultrasound scans while GV reviewed them as a third, masked observer. AU and AR helped with data management and statistics. LC critically reviewed the manuscript. All authors read and approved the final manuscript.
